# The evaluation of red blood cell folate and methotrexate levels during protocol M in childhood acute lymphoblastic leukemia

**DOI:** 10.1186/s12885-020-07422-y

**Published:** 2020-09-30

**Authors:** N. Oosterom, M. Fiocco, R. Q. H. Kloos, I. M. van der Sluis, R. Pieters, B. D. van Zelst, D. E. C. Smith, M. M. van den Heuvel-Eibrink, R. de Jonge, S. G. Heil

**Affiliations:** 1 Princess Máxima Center for Pediatric Oncology, Postbus 113, 3720 AC Bilthoven, Utrecht, The Netherlands; 2 Department of Clinical Chemistry, Erasmus MC, University Medical Center Rotterdam, Rotterdam, The Netherlands; 3 Mathematical Institute, Leiden University, Leiden, The Netherlands; 4 Department of Biomedical Data Sciences, Leiden University Medical Center, Medical Statistics, Leiden, The Netherlands; 5 Department of Pediatric Oncology/Hematology, Erasmus Medical Center-Sophia’s Children’s Hospital, Rotterdam, The Netherlands; 6 Department of Clinical Chemistry, Amsterdam UMC, Vrije Universiteit Amsterdam, Amsterdam, The Netherlands

**Keywords:** Methotrexate, Leucovorin, Acute lymphoblastic leukemia, Pediatric oncology

## Abstract

**Background:**

After High-Dose Methotrexate (HD-MTX), folinic acid rescue therapy (Leucovorin) is administered to reduce side effects in pediatric acute lymphoblastic leukemia (ALL) patients. Leucovorin and MTX are structural analogues, possibly competing for cellular transport and intracellular metabolism. We hypothesize that Leucovorin accumulates during consecutive courses, which might result in a lower MTX uptake.

**Methods:**

We prospectively measured red blood cell (RBC) folate and MTX levels during four HD-MTX and Leucovorin courses in 43 patients treated according the DCOG ALL-11 protocol with 2-weekly HD-MTX (5 g/m^2^/dose) and Leucovorin (15 mg/m^2^/dose) using LC-MS/MS. We estimated a linear mixed model to assess the relationship between these variables over time.

**Results:**

Both RBC MTX-PG and folate levels increased significantly during protocol M. MTX-PG_2–5_ levels increased most substantially after the first two HD-MTX courses (until median 113.0 nmol/L, IQR 76.8–165.2) after which levels plateaued during the 3^d^ and 4th course (until median 141.3 nmol/L, IQR 100.2–190.2). In parallel, folate levels increased most substantially after the first two HD-MTX courses (until median 401.6 nmol/L, IQR 163.3–594.2) after which levels plateaued during the 3^d^ and 4th course (until median 411.5 nmol/L, IQR 240.3–665.6). The ratio folate/MTX-PG decreased significantly over time, which was mostly due to the relatively higher increase (delta) of MTX-PG.

**Conclusion:**

These results suggest that the increase in RBC folate levels does not seem to have a large effect on RBC MTX levels. Future studies, assessing competition of Leucovorin and MTX on other cellular mechanisms which might negatively affect treatment efficacy, are necessary.

## Background

**Fig. 1:**
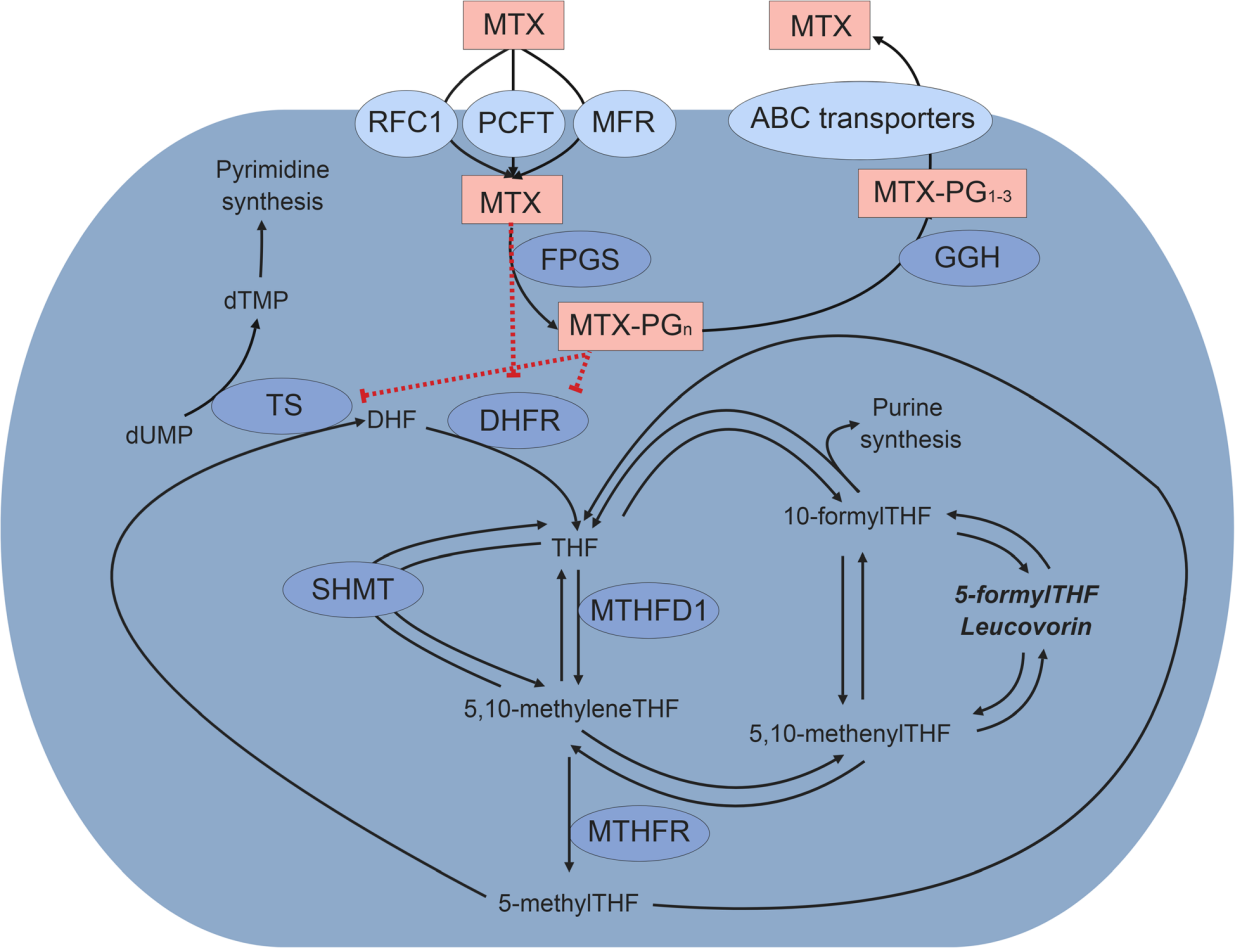
Mechanism of action MTX and LV. Overview of the folate pathway with separate folate isoforms and converting enzymes in relation to the mechanism of action of MTX. MTX enters the cell through RFC1, PCFT and MFR. MTX is then polyglutamated (−PG) by FPGS and depolyglutamated by GGH, after which MTX is exported out of the cell by ABC transporters. MTX(−PG) inhibits TS and DHFR. Leucovorin (5-formylTHF) is represented in bold / italic and bypasses the action of DHFR. Abbreviations: ABCB1 - ATP Binding Cassette Subfamily B Member 1; ABCC1–4 - ATP Binding Cassette Subfamily C Member 1–4; ABCG2 - ATP Binding Cassette Subfamily G Member 2; DHF – Dihydrofolate; DHFR – Dihydrofolate Reductase; FPGS – Folylpolyglutamate Synthetase; GGH – Gamma-Glutamyl Hydrolase; MFR – Membrane Folate Transporter; MTHFR - Methylene tetrahydrofolate reductase; MTHFD1 - Methylenetetrahydrofolate Dehydrogenase, Cyclohydrolase And Formyltetrahydrofolate Synthetase 1; PCFT – Proton-Coupled Folate Transporter; RFC1 – Reduced Folate Carrier; SHMT - Serine hydroxymethyltransferase; THF – tetrahydrofolate; TS – Thymidylate Synthase. This Figure was created using Microsoft Office Powerpoint

High-dose Methotrexate (HD-MTX) is an important component of pediatric acute lymphoblastic leukemia (ALL) treatment [1–3]. MTX is an antifolate that impairs purine- and thymidine synthesis by inhibiting the enzymes Dihydrofolate Reductase (DHFR) and Thymidylate Synthase (TS) [4]. Following HD-MTX infusions, folinic acid rescue therapy (Leucovorin – LV) is administered to reduce toxic side effects of therapy. LV is a reduced folate that bypasses the block of DHFR by MTX (Fig. 1) [5]. Leucovorin and MTX are structural analogues, possibly competing for cellular transport and intracellular pathways. Previous studies showed that most toxicity seems to occur after the first out of four HD-MTX courses, when cells have not yet been exposed to LV [6, 7]. LV restores the intracellular folate pool and might compete with MTX for cellular transport mechanisms leading to a lower uptake of MTX during consecutive HD-MTX and LV courses [8–11].

Both pediatric ALL studies [8, 10–12] and rheumatoid arthritis (RA) studies [13, 14] have shown that the administration of folate rescue therapy decreases toxicity, but might also decrease treatment efficacy – reflected by a higher risk of relapse in ALL and a higher disease activity in RA. In contrast, several studies advocate the use of higher LV doses to reduce toxicity as they were not able to show decreased treatment efficacy by LV rescue after HD-MTX [15–17]. As both treatment efficacy and toxicity are affected by the possible competition between MTX and LV, it would be of value to determine whether LV negatively affects MTX accumulation during consecutive HD-MTX and LV courses.

In this study, we prospectively determined red blood cell (RBC) folate and MTX levels longitudinally after each of the four consecutive 2-weekly 5 g/m^2^ HD-MTX courses with LV rescue in pediatric ALL patients to assess the changes in RBC folate and MTX levels over time.

## Methods

### Patient selection

Pediatric ALL patients (1–19 years) treated with HD-MTX courses according to the standard and medium risk arms of the Dutch Childhood Oncology (DCOG) ALL-11 protocol were eligible for this study. Children were newly diagnosed with ALL in the period between November 2014 and September 2018. All patients participated in the DCOG ALL-11 protocol and consented to the use of their patient data for the purpose of ALL studies (CCMO register: NL50250.078.14 / MEC-2012-287). This study was approved by the local ethics committee and according to Dutch legislation informed consent was signed by children 12–18 years old and the parents or guardians, for children < 12 years old informed consent was signed by the parents or guardians. Above the age > 18 years old the patient signed the informed consent.

### Protocol M

We studied patients prospectively during protocol M (HD-MTX phase). During protocol M, four HD-MTX infusions were administered every 2 weeks at a dose of 5000 mg/m^2^ in 24 h. Each MTX administration was combined with intrathecal triple chemotherapy in a standard dose adjusted for age (respectively, 8–12 mg MTX; 20–30 Cytosine Arabinoside; 8–12 mg Diadreson F aquosum). Folinic acid rescue (15 mg/m^2^/dose) was administered at 42, 48 and 54 h after the start of intravenous HD-MTX administration. Standard supportive care guidelines included hyperhydration (2.5–3.0 L/m^2^/day) and urine alkalinization using sodium bicarbonate (pH between 7.0–8.0). In addition, protocol M included oral 6-mercaptopurine (25 mg/m^2^ daily for 56 days). Patients had a standard 48 h hospital admission during HD-MTX courses. Plasma MTX levels were measured at 48 h (T48) after start of the HD-MTX infusion. When plasma MTX T48 levels were < 0.4 μmol/L and the patient was in a good clinical condition, the patient was discharged and the last folinic acid rescue dose was administered at home. When plasma MTX T48 levels were > 0.4 μmol/L, folinic acid was continued until plasma MTX levels were < 0.25 μmol/L. HD-MTX courses were postponed for at least 1 week when patients suffered from a severe infection, mucositis or hepatotoxicity (AST / ALT >10x upper limit of normal), when the white blood count was < 1.5 × 10^9^/L or platelets were < 50 × 10^9^/L.

### Toxicity

Toxicity was prospectively registered and graded according to the Common Terminology Criteria for Adverse Events (CTCAE) version 4.03. Registered toxicity included central neurotoxicity (ataxia, somnolence, a depressed level of consciousness, agitation, seizures and posterior reversible encephalopathy syndrome), infections and mucositis. In addition, a complete blood count, liver enzymes (alanine transaminase (ALT) and aspartate transaminase (AST)), and creatinine concentrations were measured just prior to the next high dose MTX courses. In addition, the total treatment delay in days due to extra hospital admissions and prolongation of hospital admissions due to toxicity were registered.

### Red Blood Cell (RBC) Folate and MTX-PG measurements

Samples were collected prospectively every two to three (when the course was delayed for 1 week) weeks after every HD-MTX course. Previously, it was shown that RBC MTX-PG levels did not differ significantly between samples drawn two or 3 weeks after HD-MTX [18]. In eight patients samples were also collected at start of protocol M before the first HD-MTX course. Cell pellets were harvested from centrifuged EDTA blood samples and stored at − 80 °C. RBC folate levels (non-methyl tetrahydrofolate (THF); 5-methyl THF; folic acid) were measured using liquid chromatography-tandem mass spectrometry (LC-MS/MS) as previously described [19]. The non-methyl THF pool consists of the sum of THF, 5,10-methylene THF, 5,10-methenyl THF and 5- and 10-formyl THF (Fig. 1). The sum of the total RBC folate was calculated by adding the non-methyl THF and 5-methyl THF levels. RBC MTX-polyglutamates (MTX-PG_1–5_) were measured as previously described using an LC-MS/MS method [20]. MTX-PG_1_ is freely transportable in- and out of cells, thus very variable. Therefore, only MTX-PG_2–5_ were used for analysis. Only patients having ≥3 samples available out of 4 measurements were included in our analyses. We used the total sum of RBC folate levels (non-methylTHF + 5-methylTHF + folic acid) and RBC MTX-PGs (MTX-PG_2_ + MTX-PG_3_ + MTX-PG_4_ + MTX-PG_5_) at each timepoint in our analyses. The delta of median RBC folate and MTX-PG_2–5_ levels measured between courses was calculated.

### Statistical analysis

To investigate the relationship between RBC MTX-PG and RBC folate levels a linear mixed model (LMM) was estimated. LMM accounts for the repeated measurement design of this study and takes into account that measurements belonging to the same patient are correlated. Two separate LMMs were estimated to study possible changes of RBC MTX-PG and RBC folate levels as well as the ratio RBC folate / MTX-PG over time. Due to the sample size covariates, such as erythrocyte transfusions, ALL immunophenotype or prolonged hospitalizations due to high MTX plasma levels (and increased number of LV doses), were not included in the statistical model. Possible effects of these covariates were assessed by descriptive figures. A *p*-value < 0.05 was considered statistically significant.

## Results

### Patient characteristics and toxicity

We included 43 pediatric ALL patients. They all received four HD-MTX courses including LV rescue therapy during protocol M (*n* = 172 courses). Baseline characteristics are summarized in Table 1. The median duration of protocol M was 65 days (range 56–83 days). The most frequent grade III and IV toxicities during protocol M were neutropenia (60%), leucopenia (35%) and mucositis (35%).

**Table 1:** Patient characteristics

Patient characteristics	*n* = 43
Age at diagnosis in years, *median (range)*	4.2 (1.6–17.7)
Sex, n (%)	
*Female*	18 (42%)
*Male*	25 (58%)
Immunophenotype ALL, n (%)	
*B-lineage*	38 (88%)
*T-lineage*	5 (12%)
Risk group ALL-10 protocol, n (%)	
*Standard risk*	19 (44%)
*Medium risk*	24 (56%)
Protocol M characteristics	
Duration Protocol M in days, *median (range)*	65 (56–83)
Extra hospital admissions during protocol M, *n (percentage of 172 courses)*	12 (7%)
Duration HD-MTX hospital admission in days, *median (range)*	
*After Course 1*	2 (2–13)
*After Course 2*	2 (2–5)
*After Course 3*	2 (2–8)
*After Course 4*	2 (2–6)
Toxicity during Protocol M (NCI CTC)	
Number of infections, *n (%)*	10 (23%)
Number of Erythrocyte transfusions per patient, median (range)	0 (0–3)
Number of Thrombocyte transfusions per patient, median (range)	0 (0–1)
Leukopenia, n (%)	
*Grade 1–2*	28 (65%)
*Grade 3–4*	15 (35%)
Neutropenia, n (%)	
*Grade 1–2*	17 (40%)
*Grade 3–4*	26 (60%)
Increased creatinine T48	
*Grade 1–2*	43 (100%)
*Grade 3–4*	0 (0%)
Neurotoxicity	
*Grade 1–2*	42 (98%)
*Grade 3–4*	1 (2%)
Oral Mucositis	
*Grade 1–2*	28 (65%)
*Grade 3–4*	15 (35%)

Patient characteristics of *n* = 43 pediatric acute lympoblastic leukemia patients included in this study

*NCI CTC* national cancer institute CTCAE criteria

### RBC MTX-PG and folate levels

**Fig. 2:**
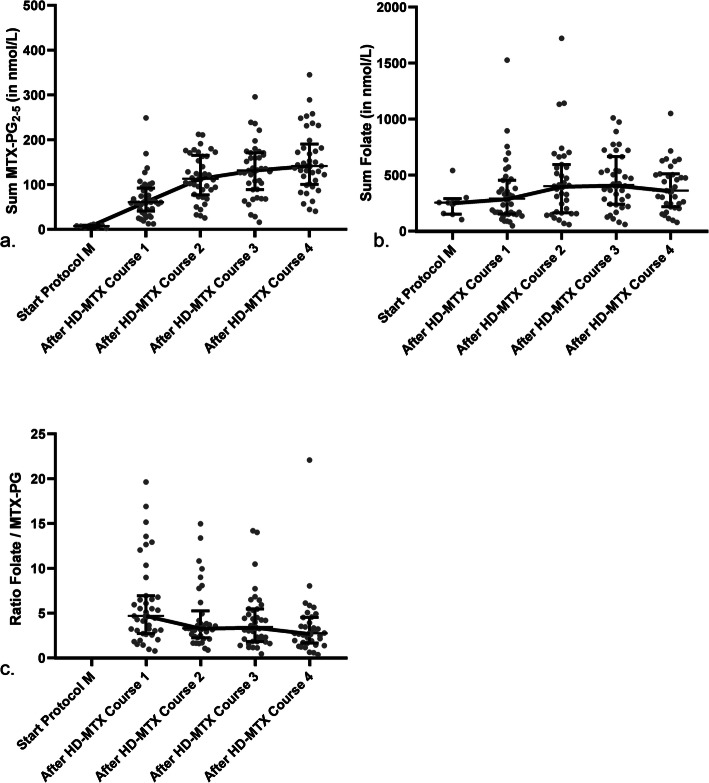
Median intracellular MTX-PG and folate levels during four consecutive courses. Measurements were performed in *n* = 8 patients at start of protocol M and in *n* = 43 patients every 2 weeks after a HD-MTX and LV course at time of qualification for the next course. Both intracellular MTX-PG (**a**) and folate (**b**) levels increased significantly during protocol M over time (*p* < 0.001). The ratio folate/MTX-PG (**c**) decreased significantly over time (*p* < 0.001). The bold line represents the median levels of patients over time. This Figure was created using Graphpad Prism version 8.3

We measured RBC MTX-PG levels at start of protocol M, which is a week *before* start of HD-MTX, in 8 patients (Table 2) and observed that low levels of MTX-PG_2–5_ were present (median 7.5 nmol/L, interquartile range (IQR) 4.2–9.2). RBC folate levels at start of protocol M, a week *before* start of HD-MTX, in these 8 patients were median 255.2 nmol/L (IQR 151.7–290.9 nmol/L). In 43 patients, RBC MTX-PG_2–5_ levels increased most substantially after the first two HD-MTX courses (until median levels of 113.0 nmol/L, IQR 76.8–165.2) as compared to after the 3rd (median 131.6 nmol/L, IQR 88.9–170.7) and 4th course (median 141.3 nmol/L, IQR 100.2–190.2), where levels plateaued (Table 2; Fig. 2). In parallel, RBC folate levels increased most substantially after the first two HD-MTX courses (until median levels of 401.6 nmol/L, IQR 163.3–594.2) as compared to after the 3rd (median 411.5 nmol/L, IQR 240.3–665.6) and 4th (median 361.5 nmol/L, IQR 217.5–511.0) course, where levels plateaued and even seemed to decrease again (Table 2; Fig. 2). For the different folate forms we observed that both 5-methylTHF and non-methylTHF levels increased over time in RBC’s (Table 2). 5-methylTHF levels increased most after the first two HD-MTX courses (until median levels of 218.3 nmol/L, IQR 91.1–386.4) as compared to after the 3rd (median 227.8 nmol/L, IQR 151.2–384.3) and 4th course (median 228.9 nmol/L, IQR 134.9–356.0), where levels plateaued (Table 2). Non-methylTHF levels increased most substantially until after the first three HD-MTX courses (until median levels of 106.3 nmol/L, IQR 63.2–208.8) as compared to after the 4th course (median 94.8 nmol/L, IQR 59.0–163.9), where levels seemed to decrease again (Table 2).

**Table 2:** Intracellular MTX-PG and eryfolate levels (*n* = 43)

RBC MTX-PG, in nmol/L	median	IQR	Delta RBC MTX-PG, in nmol/L	median	IQR
MTX-PG_2_, median (IQR)					
*Start protocol M*^*a*^	1.4	(1.2–2.4)			
*After Course 1*	8.0	(4.9–15.5)			
*After Course 2*	12.6	(6.7–23.8)	Course 2 – Course 1	+ 1.9	(−0.8–7.9)
*After Course 3*	11.0	(6.1–20.6)	Course 3 – Course 2	−1.7	(−8.7–1.5)
*After Course 4*	10.8	(6.2–25.3)	Course 4 – Course 3	+ 0.2	(−5.4–8.3)
MTX-PG_3_, median (IQR)					
*Start protocol M*^*a*^	2.5	(1.3–3.9)			
*After Course 1*	14.3	(9.4–19.6)			
*After Course 2*	26.2	(20.5–34.7)	Course 2 – Course 1	+ 11.3	(8.4–17.4)
*After Course 3*	31.1	(20.6–39.9)	Course 3 – Course 2	+ 4.2	(0.1–7.6)
*After Course 4*	33.0	(27.1–43.5)	Course 4 – Course 3	+ 3.1	(− 5.5–10.9)
MTX-PG_4_, median (IQR)					
*Start protocol M*^*a*^	2.2	(1.3–2.5)			
*After Course 1*	20.2	(11.2–28.3)			
*After Course 2*	35.8	(24.5–56.3)	Course 2 – Course 1	+ 16.6	(9.0–27.5)
*After Course 3*	46.5	31.2–59.1)	Course 3 – Course 2	+ 7.6	(3.8–13.2)
*After Course 4*	48.7	(34.0–71.4)	Course 4 – Course 3	+ 10.0	(−4.4–16.2)
MTX-PG_5_, median (IQR)					
*Start protocol M*^*a*^	1.6	(1.1–2.0)			
*After Course 1*	18.2	(8.3–22.8)			
*After Course 2*	29.9	(22.0–45.2)	Course 2 – Course 1	+ 12.8	(7.5–23.1)
*After Course 3*	36.5	(20.6–49.3)	Course 3 – Course 2	+ 6.6	(1.7–11.0)
*After Course 4*	42.9	(23.6–56.5)	Course 4 – Course 3	+ 6.2	(−8.6–13.0)
Sum MTX-PG_2–5_, median (IQR)					
*Start protocol M*^*a*^	7.5	(4.2–9.2)			
*After Course 1*	60.7	(41.0–92.0)			
*After Course 2*	113.0	(76.8–165.2)	Course 2 – Course 1	+ 43.0	(27.9–80.1)
*After Course 3*	131.6	(88.9–170.7)	Course 3 – Course 2	+ 19.4	(−0.9–33.0)
*After Course 4*	141.3	(100.2–190.2)	Course 4 – Course 3	+ 15.2	(−18.7–44.7)
RBC Folate, in nmol/L			Delta RBC Folate, in nmol/L		
5-methylTHF, median (IQR)					
*Start protocol M*^*a*^	185.7	(97.8–215.7)			
*After Course 1*	150.3	(56.6–300.6)			
*After Course 2*	218.3	(91.1–386.4)	Course 2 – Course 1	+ 82.2	(−0.6–146.3)
*After Course 3*	227.8	(151.2–384.3)	Course 3 – Course 2	+ 23.2	(−48.8–67.8)
*After Course 4*	228.9	(134.9–356.0)	Course 4 – Course 3	+ 33.6	(−71.5–107.0)
Non-methylTHF, median (IQR)					
*Start protocol M*^*a*^	42.6	(27.9–67.7)			
*After Course 1*	61.9	(39.8–132.7)			
*After Course 2*	88.1	(49.3–169.7)	Course 2 – Course 1	+ 1.8	(−24.4–82.7)
*After Course 3*	106.3	(63.2–208.8)	Course 3 – Course 2	+ 14.2	(−90.9–56.7)
*After Course 4*	94.8	(59.0–163.9)	Course 4 – Course 3	−6.9	(−80.2–54.4)
Folic acid, median (IQR)					
*Start protocol M*^*a*^	11.8	(8.0–13.7)			
*After Course 1*	11.5	(7.9–21.1)			
*After Course 2*	17.3	(10.1–22.3)	Course 2 – Course 1	−0.9	(−5.3–4.7)
*After Course 3*	13.3	(7.7–20.8)	Course 3 – Course 2	−4.3	(−11.5–1.4)
*After Course 4*	13.0	(9.0–24.8)	Course 4 – Course 3	+ 2.0	(−8.1–8.5)
Sum folate, median (IQR)					
*Start protocol M*^*a*^	255.2	(151.7–290.9)			
*After Course 1*	290.1	(152.7–453.8)			
*After Course 2*	401.6	(163.3–594.2)	Course 2 – Course 1	+ 75.9	(−51.0–194.9)
*After Course 3*	411.5	(240.3–665.6)	Course 3 – Course 2	+ 39.4	(−169.1–128.1)
*After Course 4*	361.5	(217.5–511.0)	Course 4 – Course 3	+ 20.6	(− 162.4–199.6)
Ratio RBC Folate / MTX-PG_2–5_			Delta Ratio RBC Folate / MTX-PG_2–5_		
*After Course 1*	4.7	(2.7–6.9)			
*After Course 2*	3.3	(2.2–5.2)	Course 2 – Course 1	−2.6	(−9.7–0.1)
*After Course 3*	3.4	(1.8–5.5)	Course 3 – Course 2	−0.7	(−2.4–0.9)
*After Course 4*	2.7	(1.7–4.5)	Course 4 – Course 3	− 0.6	(− 2.1–1.3)

Levels are measured in *n* = 43 patients every 2 weeks after a HD-MTX and LV course at time of qualification for the next course

Course 1: MTX-PG in *n* = 2 missing + Folate in *n* = 4 missing

Course 2: MTX-PG in *n* = 5 missing + Folate in *n* = 8 missing

Course 3: MTX-PG in *n* = 4 missing + Folate in *n* = 5 missing

Course 4: MTX-PG in *n* = 6 missing + Folate in *n* = 8 missing

^a^MTX-PG and folate levels were available in *n* = 8 patients at start of protocol M before administration of HD-MTX and LV – as these levels were only measured only in *n* = 8 patients we did not analyse these levels in the linear mixed model

*IQR* interquartile range

**Fig. 3:**
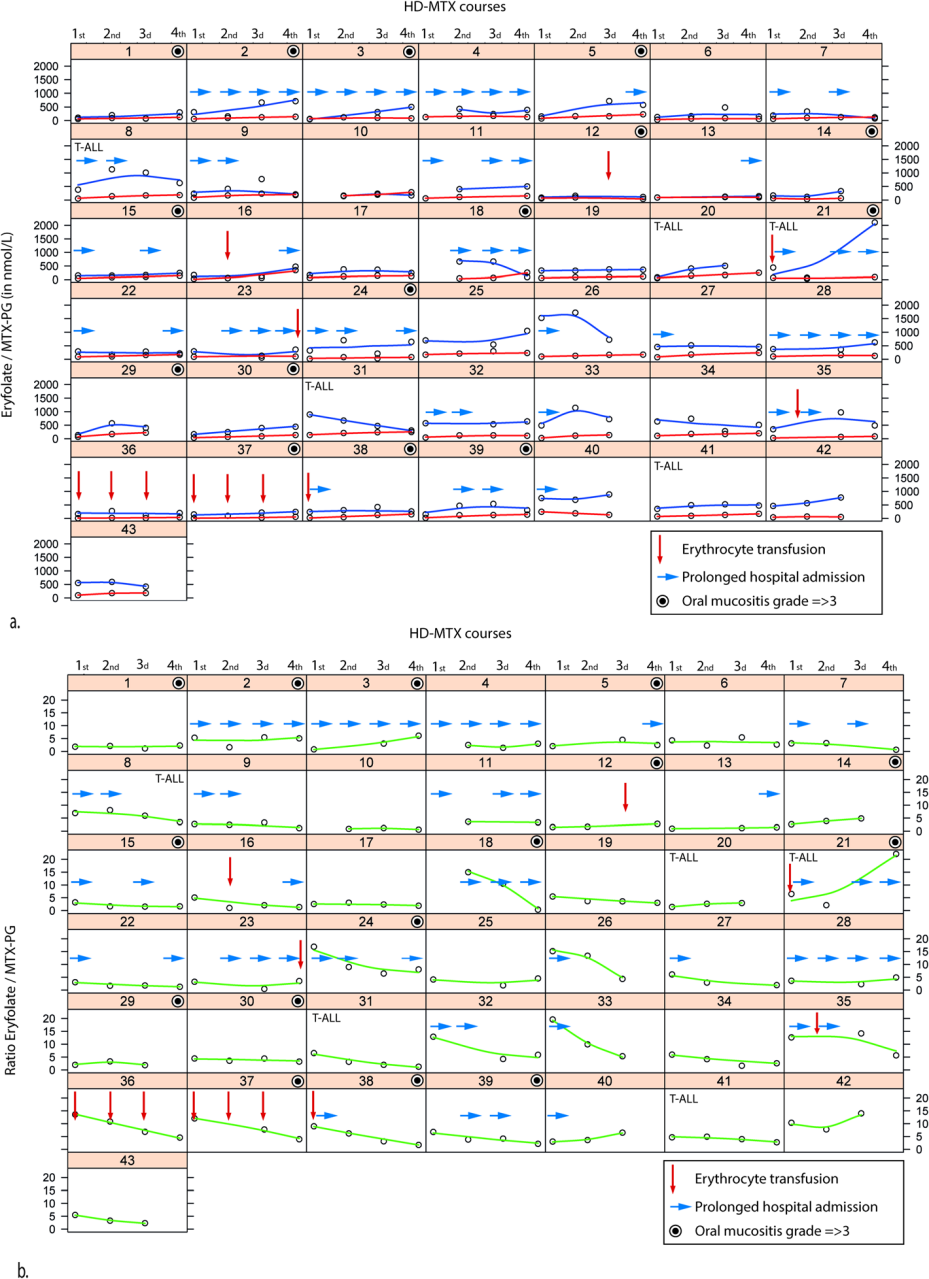
Individual MTX-PG and folate levels over time. Measurements were performed in *n* = 43 patients every 2 weeks after a HD-MTX and LV course at time of qualification for the next course. Folate levels (blue line) and MTX-PG (red line) levels in 43 individual patients are depicted over time after four HD-MTX and LV courses (**a**). The ratio folate / MTX-PG (green line) is depicted over time (**b**) after four HD-MTX and LV courses. This Figure was created using RStudio version 1.1.442

The ratio RBC folate/MTX-PG decreased over time (Figs. 2 and 3; Table 2). The decrease of the ratio was mostly due to the relatively high increase (delta) of MTX-PG levels over time, which was higher than the increase of sum folate levels (Table 2). The decrease was most prominent after the first two HD-MTX courses, after which the ratio plateaued (Table 2; Fig. 2).

A linear mixed model (LMM) to study the relation between RBC MTX-PG levels and folate levels as a function of time was estimated. The interaction term in the model was significant (*p* < 0.001), which indicates that both RBC MTX-PG as well as RBC folate levels changed significantly over time (Figs. 2 and 3; Table 2). The ratio RBC folate/MTX-PG changed significantly over time (*P* < 0.001) in a separate LMM model. Possible covariates such as erythrocyte transfusions, ALL immunophenotype or prolonged hospitalizations due to high MTX plasma levels (and increased number of LV doses) did not show large effects based on visual inspection. Results of individual patient levels are shown in Fig. 3.

## Discussion

In this study, we hypothesized that LV increases intracellular folate levels during consecutive courses, which might result in a lower uptake of MTX due to competition for cellular transport mechanisms. We showed that both RBC folate and MTX levels increased throughout four consecutive HD-MTX and LV courses and plateaued after the first two courses. This shows that the increase in RBC folate levels does not seem to have a large effect on RBC MTX levels.

Alongside with the increase of RBC folate and MTX-PG levels over time, the RBC folate/MTX-PG ratio decreased over time, which is due to the relatively higher increase of MTX-PG levels over time than to the increase of folate levels. This change of the folate/MTX-PG ratio over time is to be expected based on the higher dose of MTX (5000 mg/m^2^) compared to the cumulative dose of LV (45 mg/m^2^) administered to the patient. No decrease or less steep increase of RBC MTX was observed compared to RBC folate levels, which was expected if a large competition for cellular transport mechanisms would have been the case. True competition for cellular transport mechanisms could not be excluded as data could not be compared to patients receiving other doses of LV or no LV. An explanation for the fact that MTX-PG and LV did not seem to compete to a large extent for the use of cellular transport mechanisms could be due to passive diffusion of MTX across the membrane due to the use of high doses [21]. The restoration of the RBC folate pool could, however, still lead to a competition for binding sites of DHFR/TS or a restoration of normal DNA- and RNA- synthesis through bypassing the DHFR/TS block within cells. This could be the case in both healthy cells, leading to less toxicity during consecutive HD-MTX and LV courses, but also in leukemic blasts, leading to a decreased treatment efficacy.

We showed an increase in RBC folate levels upon consecutive HD-MTX and LV rescue courses in pediatric ALL patients. The majority of values are, however, within the range measured in the normal healthy population (RBC folate median 440.0 nmol/L, range 170.3–1164.4]) [22]. The large interindividual variability in RBC folate levels in our pediatric ALL patients are in line with the variability reported in the healthy population and may be partly explained by small differences in pre-analytical conditions, genetic variation in genes of folate transporters and metabolizing enzymes, such as the MTHFR c.677 C > T genotype, as well as differences in dietary folate intake and supplementation [22].

Median RBC MTX-PG_2–5_ levels were low at start of protocol M and accumulated until median levels of 141 nmol/L after four HD-MTX courses in 2 months. In addition, we observed very low levels of RBC MTX-PG_2–5_ levels (median level 7.5 nmol/L) in patients before start of HD-MTX treatment, presumably due to intrathecal administration of MTX in preceding chemotherapy courses, which is able to cross the blood brain barrier. MTX-PG levels after HD-MTX courses are higher compared to levels previously measured in rheumatoid arthritis (RA) patients of around 20–70 nmol/L after 2 months receiving a weekly low oral dose MTX (2.5–37.5 mg) [23, 24]. We showed that especially long chain MTX-PGs (MTX-PG_4–5_), that are associated with longer retention of HD-MTX and higher pharmacological activity, were high in our HD-MTX setting with median levels between 40 and 50 nmol/L compared to previously reported median levels between 1 and 20 nmol/L in a low dose MTX RA setting [24–26]. Previously, the same phenomenon of accumulation of especially long-chain MTX-PG’s after HD-MTX when compared to low dose MTX has been shown in leukemic blasts [27].

Previous cell line and mouse studies suggested a “selectivity” in the mechanisms of action of MTX and LV in tumor- versus in normal healthy cells [28–33]. High levels of MTX-PG accumulated in leukemia- and solid tumor cell lines, whereas only low MTX-PG levels accumulated in normal intestinal and bone marrow precursor cells [28–41]. Whether these differences in MTX-PG levels between normal and tumor cells were correlated to different levels of FPGS / GGH activity or a different cytotoxic response to MTX and/or LV has never been investigated [28–39]. This implies, that MTX-PG levels measured in our study in cell pellets from patients in clinical remission are likely lower than in their leukemic blasts. We performed measurements in red blood cells, which are expected to reflect RBC folate and MTX-PG levels in other cells. However, red blood cells do not have a nucleus nor mitochondria and therefore no active formation of DNA- or RNA structures [42]. In future studies, it would be of scientific value to determine folate- and MTX-PG levels in leukemic blasts or other nuclear blood cells such as leucocytes.

The current study had some limitations. First, we did not add covariates, such as ALL immunophenotype, the administration of erythrocyte transfusions and treatment delays to our statistical model due to the lack of power. These are factors that could potentially affect RBC folate and MTX-PG levels. It has been shown that B-ALL patients have higher RBC MTX-PG levels compared to T-ALL patients [34]. Administration of erythrocyte transfusions could potentially lead to lower RBC folate and MTX-PG levels through the introduction of exogenous erythrocytes which are naïve to MTX and LV treatment. In addition, treatment delays have been shown to not affect MTX-PG levels [18], but may lead to lower RBC folate levels. Finally, as not all additional LV infusions were registered in the medical record, we assumed that a prolonged hospitalization due to high MTX plasma levels was accompanied with more LV infusions according to protocol. Although statistical analysis was not feasible, we did not observe large effects of these covariates in our cohort through visual inspection of individual trends of MTX-PG and folate levels over time. Nevertheless, in future studies, it would be valuable to include these covariates in the analysis. In such a study, it would also be valuable to have MTX- and folate measurements at more timepoints throughout one HD-MTX course (T12; T24; T48; T72) and to measure the different folate-polyglutamate molecule levels. Finally, it would be valuable to measure these molecules in other cell types, such as the leucocyte. The major strength of this study is the prospective and consecutive measurement of combined MTX and folate levels in a homogeneously treated cohort of pediatric ALL patients throughout several HD-MTX and LV courses.

## Conclusion

In conclusion, this is the first study that measured RBC MTX and folate levels during consecutive MTX courses in ALL patients and showed that RBC MTX and folate levels increased most steeply after the first two out of four courses in red blood cells. Our results suggest that the increase in RBC folate levels does not seem to have a large effect on RBC MTX levels. In future studies, it would be valuable to study possible other cellular competition mechanisms and selective mechanisms of action of MTX and LV in leukemic blasts and healthy tissue by assessing differences in MTX polyglutamylation and FPGS/GGH activity.

## Data Availability

The dataset has been uploaded to Figshare: https://figshare.com/s/157f492ba18ce7e331d7; DOI 10.6084/m9.figshare.12909395.

## Abbreviations

ALL: Acute Lymphoblastic Leukemia
ALT: Alanine Transaminase
AST: Aspartate Transaminase
CTCAE: Common Terminology Criteria for Adverse Events
DCOG: Dutch Childhood Oncology Group
DHFR: Dihydrofolate reductase
DNA: Desoxyribonucleic Acid
GGH: Gamma-glutamyl Hydrolase
HD-MTX: High-Dose Methotrexate
IQR: Interquartile Range
FPGS: Folylpolyglutamate Synthase
LC-MS/MS: Liquid Chromatography-Mass Spectrometry
LMM: Linear Mixed Model
LV: Leucovorin
MTX-PG: Methotrexate-polyglutamate
THF: Tetrahydrofolate
TS: Thymidylate Synthase
RA: Rheumathoid arthritis
RBC: Red Blood Cell
RNA: Ribonucleid Acid
